# Chondroprotective effects of purple corn anthocyanins on advanced glycation end products induction through suppression of NF-κB and MAPK signaling

**DOI:** 10.1038/s41598-021-81384-4

**Published:** 2021-01-21

**Authors:** Hathaichanok Chuntakaruk, Prachya Kongtawelert, Peraphan Pothacharoen

**Affiliations:** Thailand Excellence Center for Tissue Engineering and Stem Cells, Department of Biochemistry, Faculty of Medicine, Chiang Mai University, Chiang Mai, 50200 Thailand

**Keywords:** Biochemistry, Biological techniques, Cell biology, Molecular biology, Diseases

## Abstract

Formation of advanced glycation end products (AGEs), which are associated with diabetes mellitus, contributes to prominent features of osteoarthritis, i.e., inflammation-mediated destruction of articular cartilage. Among the phytochemicals which play a role in anti-inflammatory effects, anthocyanins have also been demonstrated to have anti-diabetic properties. Purple corn is a source of three major anthocyanins: cyanidin-3-O-glucoside, pelargonidin-3-O-glucoside and peonidin-3-O-glucoside. Purple corn anthocyanins have been demonstrated to be involved in the reduction of diabetes-associated inflammation, suggesting that they may have a beneficial effect on diabetes-mediated inflammation of cartilage. This investigation of the chondroprotective effects of purple corn extract on cartilage degradation found a reduction in glycosaminoglycans released from AGEs induced cartilage explants, corresponding with diminishing of uronic acid loss of the cartilage matrix. Investigation of the molecular mechanisms in human articular chondrocytes showed the anti-inflammatory effect of purple corn anthocyanins and the metabolite, protocatechuic acid (PCA) on AGEs induced human articular chondrocytes via inactivation of the NFκb and MAPK signaling pathways. This finding suggests that purple corn anthocyanins and PCA may help ameliorate AGEs mediated inflammation and diabetes-mediated cartilage degradation.

## Introduction

Osteoarthritis (OA), a degenerative joint disease, is caused by disturbance of anabolism and catabolism homeostasis of articular cartilage. In 2019, the World Health Organization (WHO) ranked OA as one of the top ten diseases in developed countries and predicted that the population at risk might rise 4.5-fold within one to nine years and by 9.3-fold within ten years^1^. These predictions are consistent with the high incidence of diabetes, e.g., approximately 199 million women had diabetes in 2017, a figure that has been predicted to reach 313 million in 2040^2^. Indeed, many studies have demonstrated that the incidence and severity of OA in Diabetes mellitus (DM) patients as well as the risk of joint replacement surgery are higher in DM than non DM patients^3–6^.

Recently, much evidence has shown that hyperglycemia is one of the main causes of diabetes-mediated OA^7^. Covalent adducts of proteins, lipids or nucleic acids are formed with the aldehyde group of glucose through a non-enzymatic process called glycation that leads to an elevated formation of irreversible advanced glycation end products (AGEs). AGEs play an important role in the pathogenesis of diabetic complications contributing to OA via high levels of expression in plasma, synovial fluid and cartilage^8^. The glycation of cartilage collagen is higher than that of skin collagen^9^, affecting collagen conformation and properties such as loss of solubility and flexibility contributing to increased mechanical stress^10, 11^. Another cause of AGEs induced OA is the interaction of AGEs with the receptor for advanced glycation end products (RAGEs) on chondrocyte cell membranes^12^ which stimulates inflammation^13^ via activation of the nuclear factor NF-κB and MAPK followed by up-regulation of pro-inflammatory cytokines^14^. Pro-inflammatory cytokines, in turn, induce chondrocytes to secrete matrix metalloproteinases (MMPs) which further degrade cartilage extracellular matrix contributing to OA^15–18^.

Purple corn (*Zea mays* L.) has less starch and a lower glycemic index than their lighter colored counterparts, e.g., white corn (*Zea mays* L. var. indentata). The purple variety is considered to be less toxic and suitable for DM patients and dieters^19, 20^. Apart from hypoglycemic agents, purple corn also contains phenolic phytochemicals such as phenolic acids and flavonoids that are anti-inflammatory agents. Anthocyanins, members of the flavonoid group, are water soluble pigments which are responsible for the purple, blue and red color of plant tissues e.g., cyanidin-3-O-glucoside (C3G), pelargonidin-3-O-glucoside (Pg-3-glc), peonidin-3-O-glucoside (P3G) and malvidin-3-O-glucoside (M3G). Anthocyanins are directly absorbed and metabolized via phase I and II transformations in the liver. The main metabolite of anthocyanins in serum is protocatechuic acid (PCA)^21^.

Previous studies have shown that anthocyanins attenuate inflammation in arthritis induced rat models and IL-1β induced bovine nasal explants^22^. This phytochemical decreases type II collagen and proteoglycan degradation. Delphinidin, an aglycone anthocyanin, inhibits IL-1β induced COX-2 expression and PGE-2 production in human articular chondrocytes (HACs) by inactivation of the NF-κB pathway^23^. Similarly, sumac leaf extract, which contains anthocyanins, decreases the production of key free radical molecules released in IL-1β induced HACs^24^. A methylated anthocyanin decreases the expression of TNF-α, IL-1β and MMPs in OA rats via the inhibition of NF-κB^25^.

Anthocyanins have been demonstrated to be chondroprotective, having anti-inflammatory properties and downregulating pro-inflammatory cytokines and MMPs, in both in vivo and in vitro models of previous studies^26–30^. However, the effect of anthocyanins on AGEs induced OA has not been extensively studied. The objectives of this study were to determine the chondroprotective effects of purple corn extract on AGEs induced cartilage and to further explore the molecular mechanisms of purple corn anthocyanins and their metabolite on AGEs induced primary HACs.

## Results

### Anthocyanin content in purple corn extract (*Zea mays* L.)

**Figure 1:**
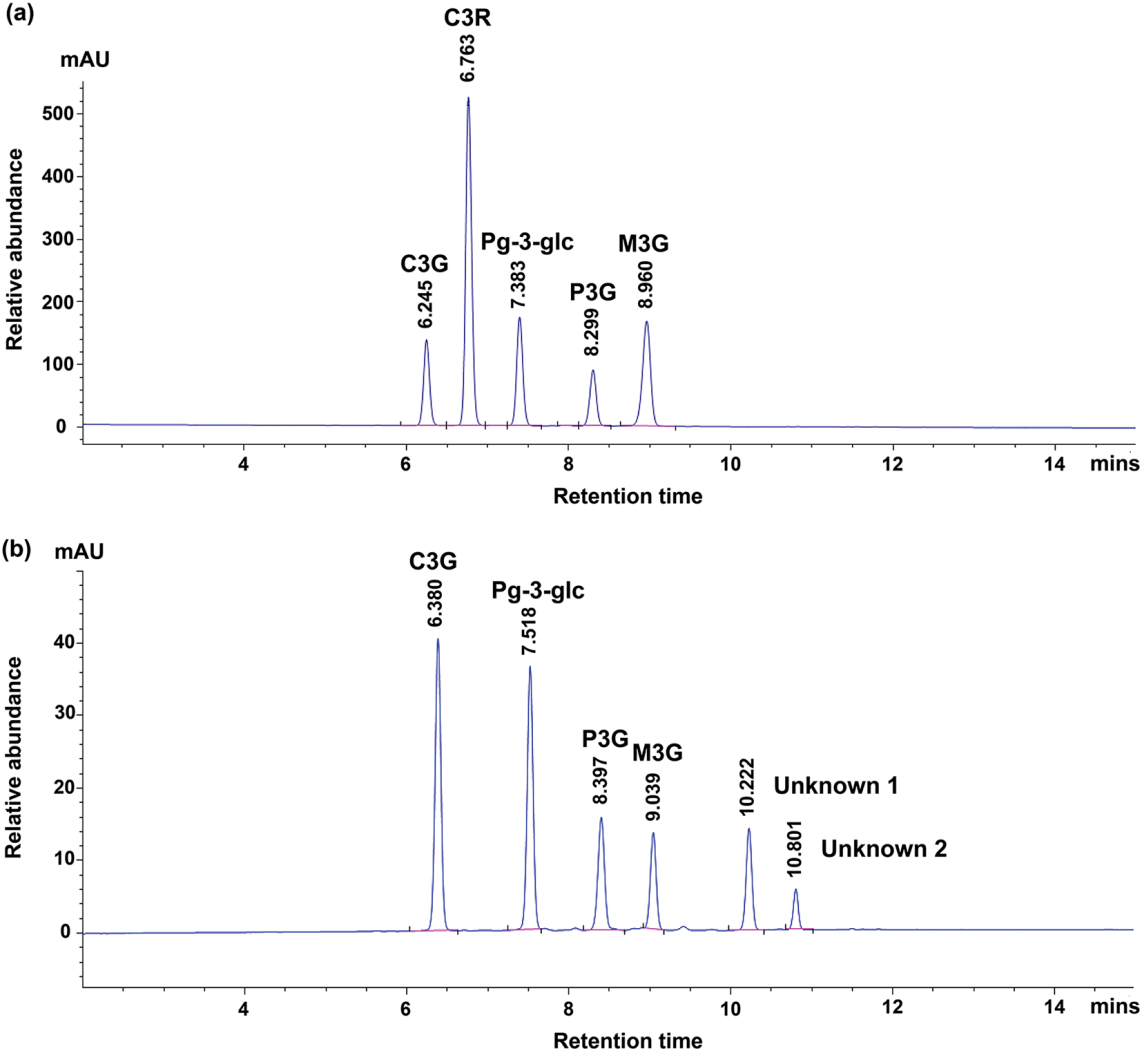
HPLC**-**chromatograms of anthocyanin standards (**a**) and purple corn extract (**b**). The numbers shown in the chromatograms are the retention times of the compounds.

The anthocyanin content in methanolic purple corn extract was analyzed using HPLC by comparing their retention times with authenticated anthocyanin standards (100 μg/ml of cyanidin-3-O-glucoside chloride (C3G), cyanidin-3-O-rutinoside chloride (C3R), pelargonidin-3-O-glucoside (Pg-3-glc), peonidin-3-O-glucoside chloride (P3G), malvidin-3-O-glucoside (M3G) and cyanidin chloride) (Fig. 1a). The chromatogram showed the anthocyanin content in methanolic purple corn extract contained C3G, Pg-3-glc, P3G and M3G (Fig. 1b) which correspond to the anthocyanin standards. However, two unidentified compounds, designated as unknowns 1 and 2, were also found in the chromatogram. To find the total anthocyanin content needs to be compared to HPLC-chromatograms of anthocyanin standards, thus both unknown 1 and 2 compounds are not included in this content. However, the proportion of unknowns can be represented by the percentage of area under HPLC curve. Linear regression equations of the calibration curve which determined the anthocyanin content in the extract showed that the total anthocyanin content in the extract was 137.28 ± 0.84 μg/g of crude extract. The amounts of anthocyanins, from high to low, were C3G (53.39 ± 0.54 μg/g of crude extract), followed by Pg-3-glc (34.21 ± 0.13 μg/g of crude extract), P3G (33.18 ± 0.12 μg/g of crude extract) and M3G (16.50 ± 0.05 μg/g of crude extract). Our findings are in concordance with previous reports^31^ which reported that C3G is the most abundant anthocyanin in purple corn.

### Effect of purple corn extract on glycosaminoglycan release by AGEs treated cartilage

**Figure 2:**
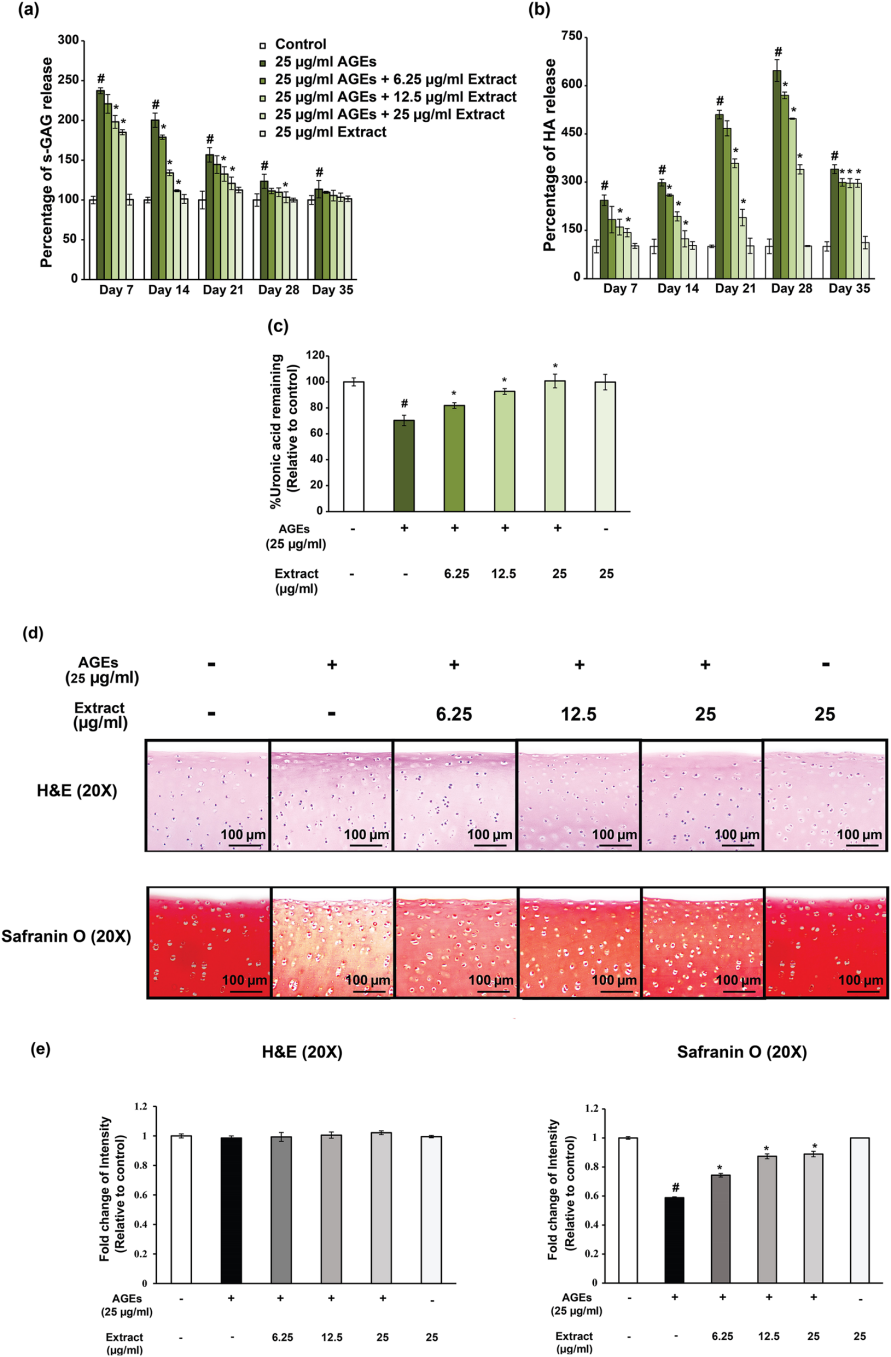
The release of s-GAG and HA in culture media of cartilage explants which treated with AGEs alone or co-treated with purple corn extract (6.25–25 μg/ml) for 35 days. Culture media at day 0, 7, 14, 21, 28 and 35 from each group was collected and levels of s-GAG (**a**) and HA (**b**) were measured. Cartilage was predigested with 10 units/ml papain and analyzed for uronic acid content (**c**). Cartilage sections were stained with H&E staining and Safranin O (**d**) and the intensity of the stained sections were analyzed using ImageJ software (**e**). Values are expressed as the average mean ± S.D. of triplicate experiments. #*p* < 0.05 compared to untreated group; *p < 0.05 compared to AGEs treatment alone.

The results showed that AGEs significantly increased the release of sulfated glycosaminoglycan (s-GAG) in the culture media when compared to the untreated group. The highest s-GAG release in the AGEs treated group was observed at day 7 and gradually declined from day 14 to day 35. On the other hand, the release of s-GAG significantly declined from day 14 to day 21 in the samples with purple corn extract co-treated with AGEs. There was no difference in s-GAG release in the group with a high dose of purple corn extract (25 μg/ml) treated group and the untreated group (Fig. 2a). The release of hyaluronic acid (HA) in the AGEs-treated media gradually increased from day 7 to day 35 compared to the untreated group. The highest HA release in the AGEs-treated group was observed at day 28, then declined through day 35. The release of HA in the group co-treated with purple corn extract in the presence of AGEs significantly declined from day 7 to day 28 compared to the AGEs treated group in a dose dependent manner. However, HA level in the media of the high dose of purple corn extract (25 μg/ml) treated group was not different from the untreated group (Fig. 2b).

Uronic acid content in the cartilage tissues treated with AGEs or co-treated with purple corn extract at day 35 was analyzed. The results showed that AGEs significantly decreased uronic acid in porcine cartilage tissues compared to the untreated group. On the other hand, the level of uronic acid remaining in the porcine cartilage tissues when co-treated AGEs with purple corn extract (6.25–25 μg/ml) was higher than in the group treated with AGEs alone in a dose dependent manner. The effect of treatment with 25 μg/ml extract alone on uronic acid content was not different from the untreated group (Fig. 2c).

Histological analysis of the effect of purple corn extract on matrix s-GAG accumulation was performed. Porcine cartilage tissues at day 35 were sectioned and stained with Hematoxylin and eosin (H&E) or Safranin O for cell morphology and s-GAG accumulation, respectively. The H&E staining analysis demonstrated that neither AGEs (25 μg/ml) nor co-treatment with purple corn extract (6.25–25 µg/ml) affected the chondrocyte morphology or extracellular matrix architecture (Fig. 2d,e). The Safranin O staining showed that treatment with AGEs decreased s-GAG accumulation in matrix compared to the untreated sections. Co-treatment with purple corn extract reversed the effects of AGEs (Fig. 2d,e). Safranin O staining intensity of the 25 μg/ml purple corn treated section was not different from the untreated section.

### Effect of purple corn anthocyanins and PCA on AGEs induced matrix metalloproteinases expression in human articular chondrocytes (HACs)

**Figure 3:**
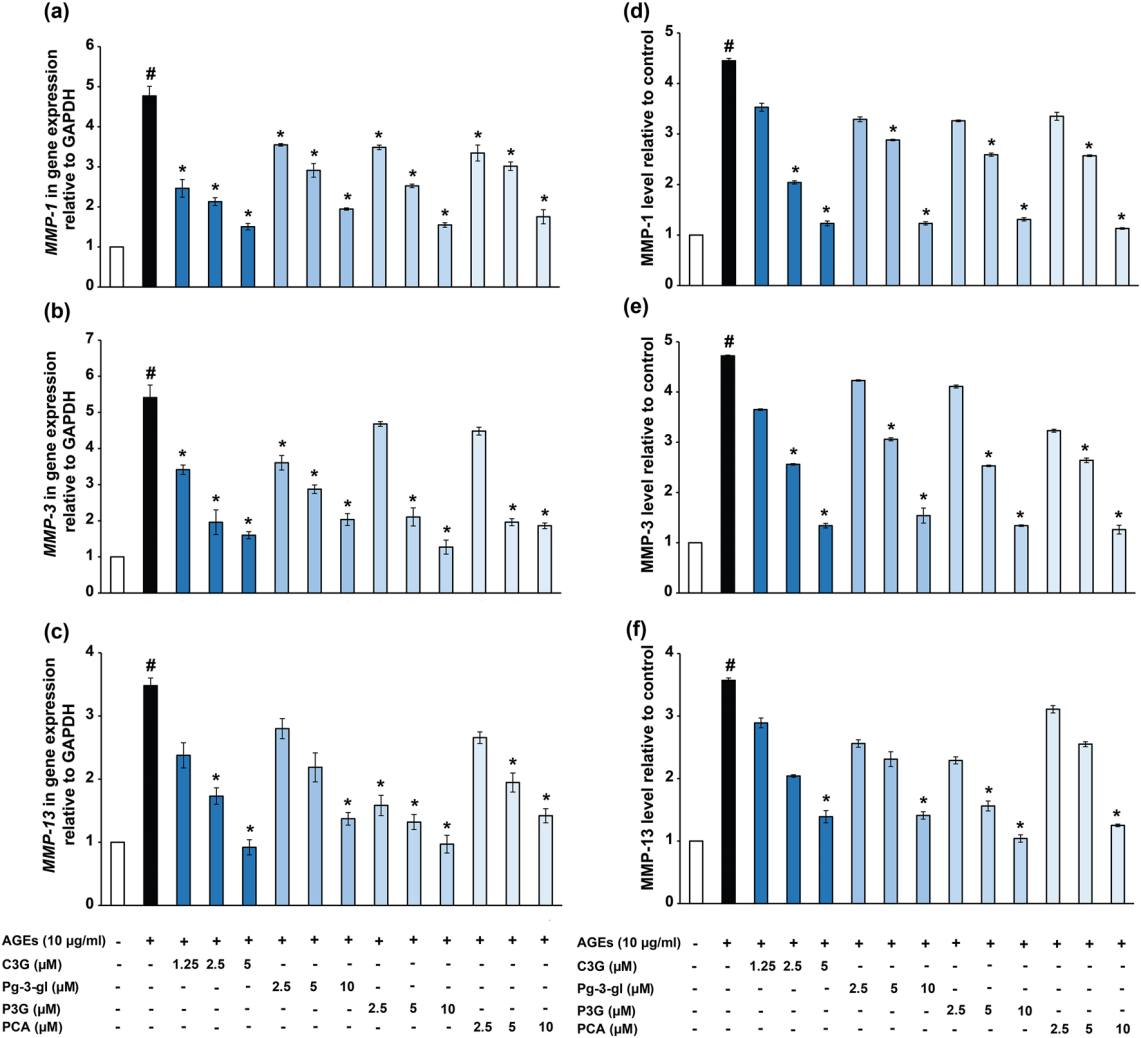
Effect of C3G, Pg-3-glc, P3G and PCA on AGEs induced HACs. The mRNA expression of *MMP-1* (**a**), *MMP-3* (**b**), *MMP-13* (**c**) and protein expression of MMP-1 (**d**), MMP-3 (**e**) and MMP-13 (**f**) were analyzed after 24 h treatment by quantitative real-time PCR and ELISA assay, respectively. Data are expressed as the average mean ± S.D. of triplicate experiments. #*p* < 0.05 compared to untreated group, *p < 0.05 compared to AGEs treated alone.

The mRNA expression levels of *MMP-1*,*-3* and *-13* in HACs were determined by quantitative real-time PCR. The treatment of HACs with AGEs (10 µg/ml) or co-treatment with C3G, Pg-3-glc, P3G and PCA for 24 h was followed by mRNA extraction and qPCR analysis. The results showed that 10 µg/ml of AGEs significantly induced the expression of *MMP-1*, *-3* and *-13* when compared to the untreated HACs. The co-treatment of AGEs with C3G (1.25–5 µM), Pg-3-glc (2.5–10 µM), P3G (2.5–10 µM) and PCA (2.5–10 µM) significantly decreased the expression of *MMP-1*, *-3* and *-13* in a dose dependent manner (Fig. 3a–c). Moreover, the secretory MMP-1, -3 and -13 levels in cultured media were increased when HACs were induced with AGEs compared to the untreated group. Co-treatment with C3G, Pg-3-glc, P3G and PCA significantly decreased secretory MMPs levels in a dose dependent manner (Fig. 3d–f).

The inhibitory activity on AGEs mediated MMPs expression was determined by the half maximum inhibitory concentration (IC_50_) of each compound. The approximate IC_50_ values showed that C3G had greater potency in inhibiting gene expression and MMPs secretion than Pg-3-glc, P3G and PCA (Table 1).

**Table 1:** Anthocyanins concentration for approximate 50% inhibition of MMPs gene expression (a) and protein expression (b).

	Anthocyanins (μM)			Metabolite (μM)
	C3G	Pg-3glc	P3G	PCA
**(a)**				
**50% reduction of gene expression (IC**_**50**_**)**				
*MMP-1*	2.8 ± 0.7	7.7 ± 0.9	6.6 ± 0.9	7.3 ± 0.9
*MMP-3*	2.7 ± 0.8	6.8 ± 0.8	5.9 ± 0.8	6.3 ± 0.7
*MMP-13*	3.0 ± 0.9	7.8 ± 0.9	4.8 ± 0.7	7.5 ± 0.9
**(b)**				
**50% reduction of MMPs expression (IC**_**50**_**)**				
MMP-1	3.1 ± 0.9	6.7 ± 1.0	6.6 ± 0.9	6.3 ± 1.0
MMP-3	3.1 ± 0.9	7.5 ± 1.0	6.7 ± 0.9	6.2 ± 0.9
MMP-13	3.5 ± 1.0	7.7 ± 0.9	5.7 ± 0.8	7.9 ± 1.0

### Effect of purple corn anthocyanins and PCA on AGEs induced NF-κB and MAPK signaling pathways in human articular chondrocytes (HACs)

**Figure 4:**
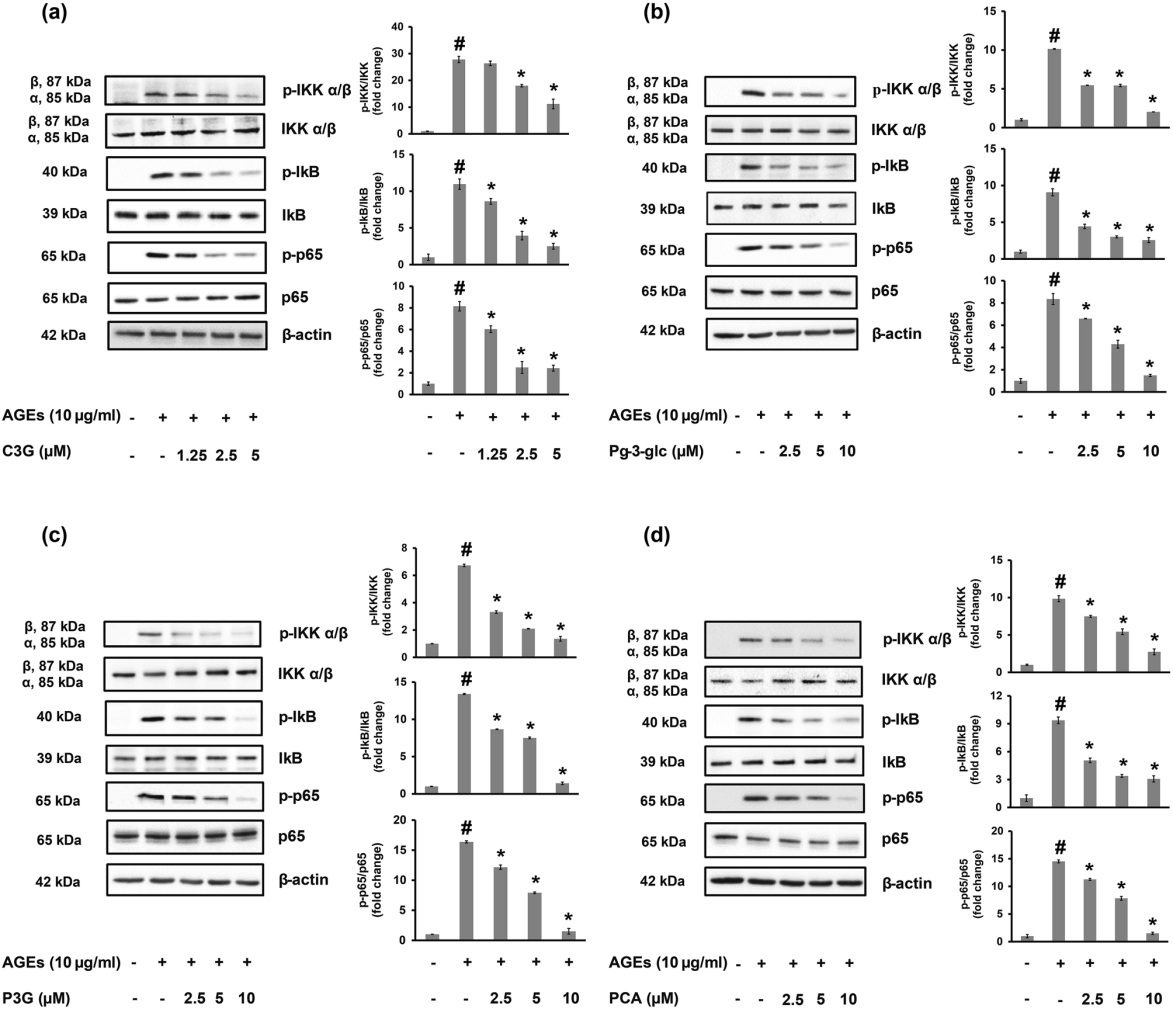
Effect of C3G, Pg-3-glc, P3G and PCA on AGEs activated NF-κB signaling pathways in HACs. The phosphorylation levels of IKK, IκB and p65 were measured by western immunoblotting analysis. The density of each band was analyzed using TotalLab TL120. Data are expressed as the average mean ± S.D. of triplicate experiments. #*p* < 0.05 compared to untreated group; *p < 0.05 compared to AGEs treated alone.

The results showed that all of the compounds decreased the phosphorylation levels of IKK, IκB and p65 when compared to the AGEs alone treatment in a dose dependent manner (Fig. 4).

**Figure 5:**
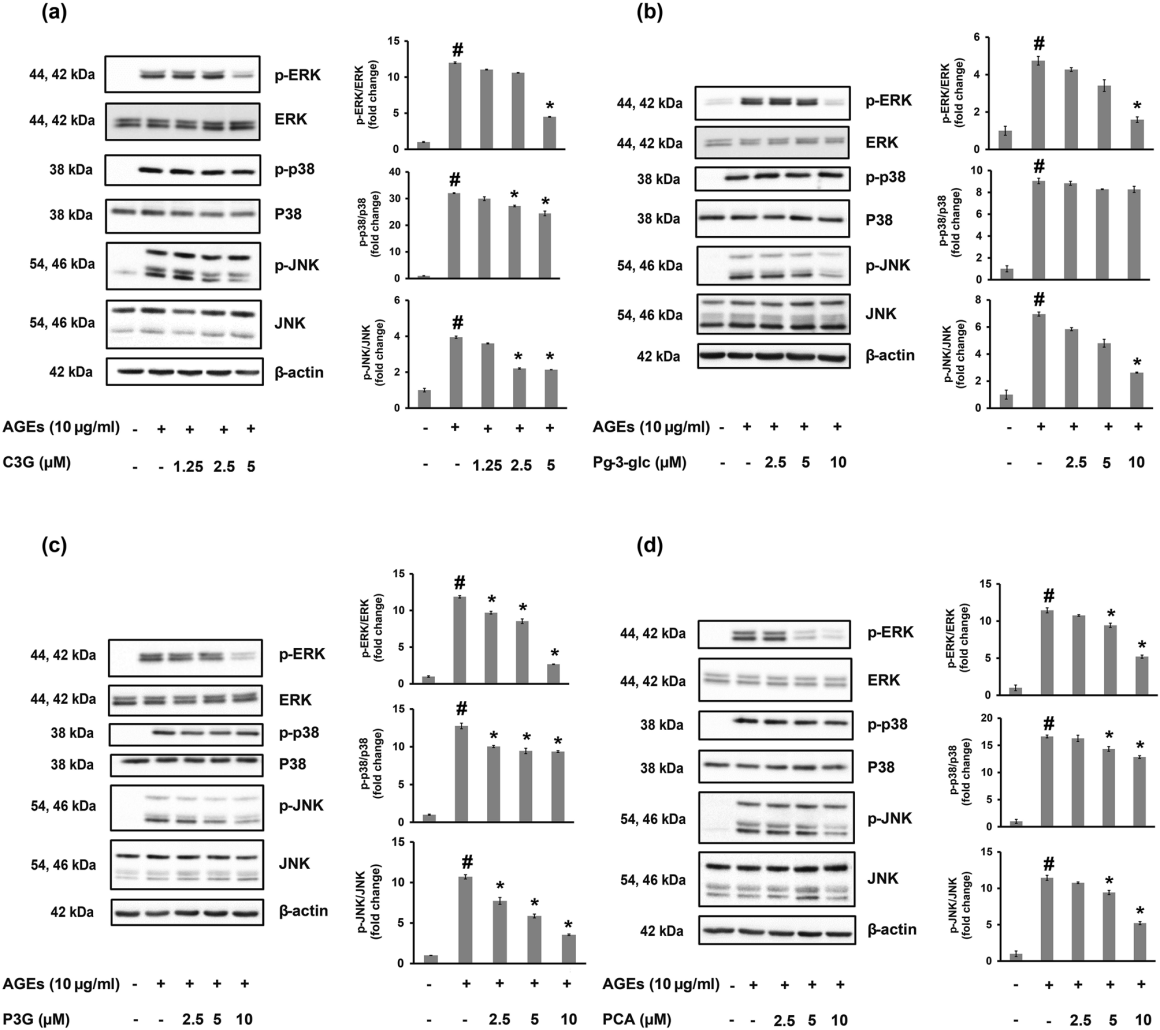
Effects of C3G, Pg-3-glc, P3G and PCA in AGEs activated MAPK signaling pathways in HACs. The phosphorylated level of ERK, p38 and JNK were measured by western immunoblotting analysis. The density of each band was analyzed using TotalLab TL120. Data are expressed as the average mean ± S.D. of triplicate experiments. #*p* < 0.05 compared to untreated group; *p < 0.05 compared to AGEs treated alone.

The effects of anthocyanins and PCA on MAPK signaling activation were also investigated. The results showed that C3G, P3G and PCA significantly reduced the phosphorylation levels of ERK, p38 and JNK compared to the AGEs treatment alone. The Pg-3-glc also significantly inhibited ERK and JNK activation but showed no effect on the phosphorylation level of p38 (Fig. 5).

## Discussion

The results of the analysis of anthocyanin content in this study are in agreement with previous studies which indicate that the major anthocyanins found in purple corn extract are cyanidin-3-glucoside (C3G), pelargonidin-3-glucoside (Pg-3-glc) and peonidin-3-O-glucoside (P3G)^32–35^. The two unknowns on the HPLC chromatogram, based on references in previous studies^31, 36, 37^ in which the same column and the HPLC method were used, are probably pelargonidin-3-(6″-malonylglucoside) and peonidin-3-(6″-malonylglucoside) according to the retention time.

In the cartilage degradation model, AGEs significantly induced s-GAG and HA release from porcine cartilage which indicates matrix glycosaminoglycans degradation. Interestingly, different patterns of s-GAG and HA release in the presence of AGEs were found. The highest release of s-GAG was on day 7 followed by the peak release of HA at day 28 followed by a decline. There are many factors contributing to AGEs induced s-GAG and HA degradation patterns. First, HA and s-GAG are substrates of a different class of enzymes. Hyaluronidases are a family of enzymes that are notably responsible for degrading HA^38^, whereas s-GAGs, both the free form and the intact form, on aggrecan fragments are the product of various types of enzymes, including aggrecanases, hyaluronidases and MMPs^39^. Second, the difference in the assembling pattern of the aggrecan and HA molecules in the cartilage matrix makes both of aggrecan core protein and glycosaminoglycan chains exposed to specific degrading enzymes more than the HA chain, as the cleavage sites in the latter are covered with aggrecan and other extracellular matrix^40^. Third, GAGs are a major component of the cartilage extracellular matrix, appearing in higher amounts than HA, which leads to a high turnover rate after degradation. The s-GAGs and HA are gradually released from the AGEs treated cartilage explants, so at the end of the explant culture (day 28 and day 35), s-GAG and HA secretion in response to AGEs was not dose dependent due to the low level of glycosaminoglycan remaining in the cartilage matrix. Histological analysis of AGEs treated cartilage correlates with the chemical analysis which demonstrated a reduction of s-GAG accumulation of AGEs treated cartilage. These results are in line with a previously study which reported high accumulation of AGEs in degraded tibial plateau cartilage of OA patients^41^. In the presence of purple corn extract, AGEs induced cartilage degradation was significantly attenuated in a dose dependent manner.

The study of bioavailability of anthocyanin after ingestion and metabolism in humans stated that C3G, Pg-3-glc, P3G and their metabolite, PCA, were found in circulating blood^42, 43^, thus, these compounds were further investigated in chondrocytes. AGEs has previously been shown to up-regulate pro-inflammatory cytokines^44^ and protease enzymes^45, 46^ in HACs. Our study of AGEs induced HACs showed that purple corn anthocyanins and PCA have the ability to reduce the gene and protein expression of MMPs. The half maximal inhibitory concentration (IC_50_) showed that among the purple corn anthocyanins, C3G has the highest efficacy in decreasing both gene and protein expression of MMP-1, -3 and -13.

The AGEs mediated activation of MAPK and the NF-κB signaling pathways in primary HACs has been previously reported. Several studies have described the role of the MAPK signaling pathway in MMP synthesis^44, 47, 48^ and how activation of ERK1/2 and p38 plays a role in abnormal chondrocytes in osteoarthritis^49, 50^. Additionally, NF-κB is the protein complex that controls inflammatory cytokines production^44^. One of two pivotal kinases, IκB kinase (IKK) α, regulates total collagenase and MMP-13 activities both in human OA and in differentiating primary murine chondrocytes^51^. Thus, NF-κB is an essential transcription factor in the regulation of matrix lysis enzymes in chondrocytes including MMP-1, 3 and 13^52^. Our results demonstrate that pretreatment with purple corn anthocyanins (C3G, Pg-3-gl, and P3G) and PCA decreases expression of the AGEs induced NF-κB and MAPK signaling pathway. Interestingly, the decrease in the phosphorylation of IKK, IκB and p65 by anthocyanins and PCA was found to be greater than the decrease in the phosphorylation of JNK, p38 and ERK.

Our study showed that among purple corn anthocyanins, C3G exhibits the highest inhibitory potency on AGEs induced MMPs expression and inflammatory response. A possible explanation is that the anti-radical activity of C3G is superior to those of P3G and Pg3-glc^53^. In addition to the NF-κB and MAPK pathways, other AGEs mediated cellular responses are activated through phosphatidyl inositol 3-OH kinase (PI3K)^54^ and NADPH oxidase^55, 56^. The activation of both those enzymes results in increased formation of reactive nitrogen species (RNS)^13^. Additionally, previous studies have demonstrated that AGEs-RAGE interaction mediates NF-κB activation and is followed by expression of iNOS^57^ and increased ONOO- formation in chondrocytes^58^. The excessive production of RNS leads to cell death and articular cartilage matrix degradation^59, 60^. C3G has previously been shown to have stronger anti-radical activity over P3G and Pg-3-glc which corresponds to the number and position of hydroxyl groups in position 3′,4′ of the anthocyanin B-ring^53, 61, 62^. To date there is no recommended dosage of purple corn anthocyanin for human health benefits. Consumption of a 500 mg oral bolus dose of ^13^C-labelled C3G showed that Cmax was 0.14 µM in plasma^43^. Regarding pharmacokinetics of C3G in human from previous study, the effective dose of C3G (5 µM) in our study corresponds to ~ 18 g of C3G/day/person. The reported daily intake of anthocyanin in United States was 12.5 mg/day/person^63^, thus, 0.07% of the dose in our study corresponds to the human daily intake.

PCA is produced after ingestion of an anthocyanin-rich diet and distributes through blood circulation to body tissues^64–68^ where it remains longer than its parent compound^69^. The anti-inflammatory effect of PCA on AGEs induced chondrocytes in this study and the anti-oxidant activity which has been previously reported^70, 71^ suggest that PCA may be the primary active compound mediating the beneficial effects of consumption of anthocyanins.

**Figure 6:**
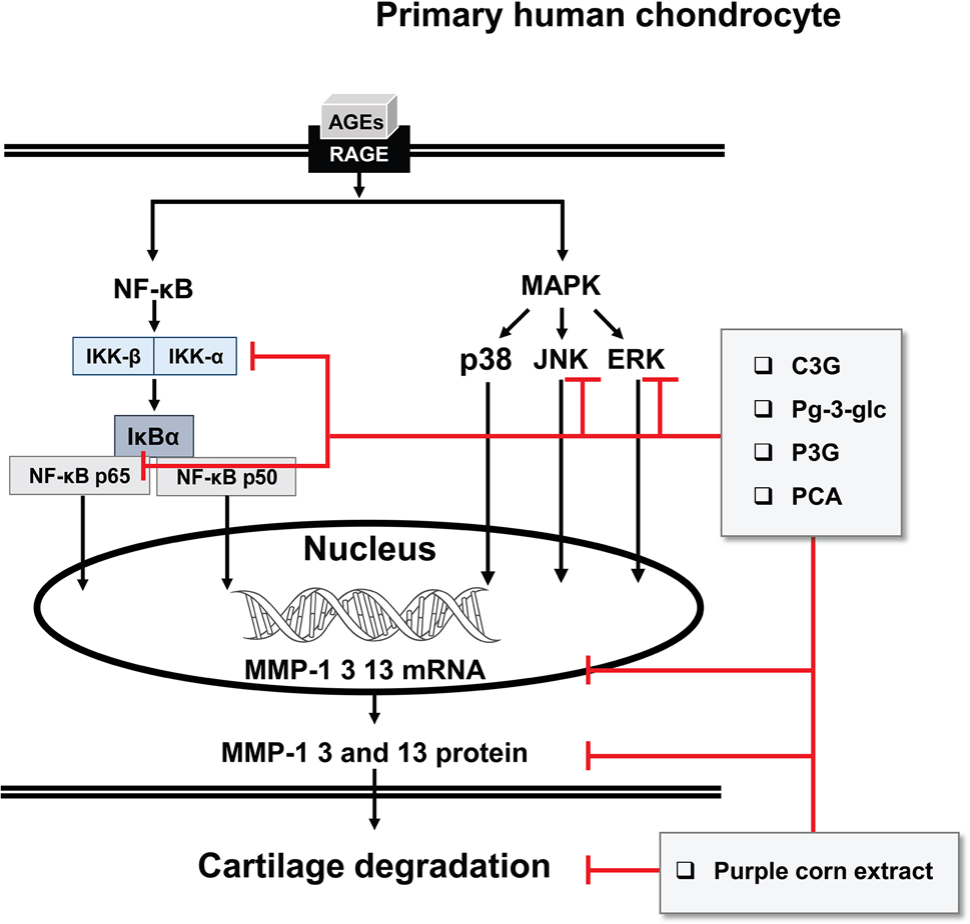
Inhibitory effects of purple corn anthocyanins and purple corn extract on advanced glycation end products induced inflammation in chondrocytes. C3G, cyaniding-3-O-glucoside; Pg-3-glc, pelargonidin-3-O-glucoside; P3G, peonidin-3-O-glucoside; PCA, protocatechuic acid.

Anthocyanins were previously showed to have in vitro anti-inflammatory effect on IL-1β induced chondrocytes^23, 29^ and in vivo chondroprotective effect in arthritis animals^22, 72^. Our study showed anthocyanins from purple corn and PCA display anti-inflammatory effects on AGEs induced chondrocytes (Fig. 6), suggesting that consumption of purple corn could be a candidate dietary supplement with benefits for OA patients with DM as well as for individuals with asymptomatic OA together with DM. This finding provides basic evidence to help guide future studies, e.g., studies of the mechanisms of anthocyanins and PCA on other AGEs-RAGEs signaling and in vivo modeling to confirm the bioactivity.

## Methods

### Purple corn extract preparation

Purple corn (*Zea mays* L.) was planted and harvested in 2017 from San Pa Tong District, Chiang Mai, Thailand. The dried purple corn kernels were ground and extract was obtained by immersion in 0.1 N HCl and methanol (20:80) at room temperature for 24 h. The ratio of purple corn to methanol was 1:10. The extract was then evaporated *in vacuo* at 40–45 °C and freeze-dried to obtain dry extract.

The anthocyanin content of the methanolic purple corn extract was analyzed using an Agilent 1260 series HPLC (Agilent Technologies Ltd., Santa Clara, CA, USA), equipped with a binary pumping system. Using the Chromadex method with minor modifications, 10 µl samples were injected into a Zorbax Eclipse Plus C18 column (4.6 × 100 mm, particle size 3.5 µm). The mobile phase consisted of water/formic acid (A) (90:10, v/v) and acetonitrile (B). The gradient phase was accomplished at 35 °C as follows: initial time, 4% solution (B); 8 min, 15% solution (B); 24 min, 80% solution (B); 30 min, 4% solution (B) at a flow rate of 1.0 ml/min. The main compounds of the extract were analyzed at a wavelength of 530 nm. Authentic standard anthocyanin, i.e., cyaniding-3-O-glucoside (C3G), Cyanidin-3-O-rutinoside chloride (C3R), peonidin-3-O-glucoside (P3G), pelargonidin-3-O-glucoside (Pg-3-glc) and malvidin-3-O-glucoside (M3G), were purchased from Sigma-Aldrich (St. Louis, MO, USA). The peak areas of the extract were calculated using calibration curves constructed by injecting the reference standard concentration range of 0–50 µg/ml.

### Porcine cartilage explant preparation

Porcine articular cartilage from the metacarpophalangeal joints of 6–8 months old pigs was dissected into 25 cm^3^ discs. The cartilage was then incubated in Dulbecco's Modified Eagle Medium (DMEM) with penicillin/streptomycin 5% (v/v) and fetal calf serum (FCS) for 30 min at 37 °C and 5% CO_2_ in a petri dish. After that, the cartilage was incubated in refreshed medium for 24 h to ensure sterility. Then 30–35 mg portions of cartilage were placed into wells of 24 well-plates and cultured at 37 °C and 5% CO_2_ in DMEM.

All the protocols using porcine cartilage were in accordance with the guideline and approved by the animal ethic committee, Faculty of Medicine, Chiang Mai University. All experimental protocols were approved by the animal ethic committee, Faculty of Medicine, Chiang Mai University.

To investigate the effect of purple corn extract on AGEs induced porcine cartilage degradation, the cartilage discs (30–35 mg) were co-treated with 25 µg/ml of AGEs (Merck Millipore, Burlington, MA, USA) and various concentrations of either purple corn extract (6.25–25 µg/ml) or PCA (6.25–25 µg/ml) for 35 days. Culture media was collected and replaced on day 0, 7, 14, 21, 28 and 35 for measurement of HA and s-GAG release. The cartilage discs at day 35 were collected for papain digestion and histological analysis.

### Measurement of s-GAG levels

The s-GAG in the conditioned culture medium for all culture conditions was measured by dimethyl-methylene blue (DMMB) assay^73^. Briefly, 200 µl of DMMB was added to 50 µl of standard chondroitin sulfate-C (0–40 µg/ml) to culture medium. The complex of DMMB and s-GAG was measured using a Multiskan Ex microplate reader (Thermo Scientific, Waltham, MA, USA) at 520 nm. The amounts of s-GAG were determined from the standard curve and presented as the percentage of s-GAG release. The formula for calculating s-GAG release percentage was: [s-GAG of each day (day 7, 14, 21, 28 or 35) − s-GAG of day 0 × 100)]/s-GAG of day 0.

### Measurement of HA levels

The release of HA from cartilage discs to the culture media was determined using the competitive enzyme linked immunosorbent assay (ELISA)^74^. Umbilical cord HA (100 µl/well) in the buffer was coated onto each well of 96-well microplates (Maxisorp Nunc, Waltham, MA, USA) at 4 °C and left overnight. Then 1% (w/v) BSA (Sigma-Aldrich, St. Louis, MO, USA) in PBS was used to block the plate for 1 h. After washing with PBS, 100 µl of the biotinylated hyaluronan, binding proteins (B-HABPs) (1:100) and a standard competitor (HA Healon: range 0–10,000 ng/ml) or a sample mixture was added to the plates which were then incubated for 1 h at room temperature. After that, the plates were washed before adding peroxidase-mouse monoclonal anti-biotin (100 µl/well; 1:2000) and incubated for another 1 h at room temperature. The plates were then washed again before adding the peroxidase substrate (100 µl/well). The reaction was allowed to continue for 5–10 min at 37 °C to allow color development then the reaction was stopped by adding 50 µl of 4 M H_2_SO_4_ per well. The absorbance ratio at 492/690 nm was measured using a microtiter plate reader (MULTISKAN Ex, Thermo Scientific, Waltham, MA, USA). The amount of HA was determined from the standard curve. The percentage of HA released was calculated using the following formula: HA of each day (day 7, 14, 21, 28 or 35) − HA of day 0 × 100)]/HA of day 0.

### Measurement of uronic acid remaining in cartilage discs

The papain-digested cartilage discs at day 35 were analyzed for uronic acid (UA) by m-hydroxydiphenyl colorimetric assay^75^. Standard glucuronic acid lactone (0–40 µg/ml) or diluted samples were added together with 300 µl of concentrated sulfuric acid-borate reagent. Incubation was carried out for 15 min at 100 °C. After that, the discs were cooled on ice before adding 12 µl of carbazole solution and incubation for a further 15 min at 100 °C. The color of the reaction was analyzed at 540 nm using a microtiter plate reader (MULTISKAN Ex, Thermo Scientific, Waltham, MA, USA). The level of UA was determined by a standard curve. The percentage of uronic acid remaining was calculated using the following formula: (uronic acid content (g) × dilution factor × dry weight (g) / uronic acid content in control cartilage) × 100.

### Histological analysis by Hematoxylin and eosin (H&E) and Safranin O staining

Cartilage samples were fixed in 4% paraformaldehyde and embedded in wax, then were then cut into 5 mm thick sections perpendicular to the articular cartilage surface. The sections were evaluated for tissue morphology and s-GAG accumulation by staining with Hematoxylin and eosin (H&E) and Safranin O, respectively, using a light microscope and photographed (Zeiss Axio Scope A1, Gottingen, Germany) at 200× magnification. The quantification of staining intensity was performed by ImageJ software available at the national institute health of (USA), downloadable from https://imagej.nih.gov/ij/download.html as for December 2020.

### Isolation and culture of chondrocytes

Non-OA joint material were obtained following informed consent from patients at Maharaj Nakorn Chiang Mai Hospital after obtaining approval for the study form the Research Ethic committee of Faculty of Medicine, Chiang Mai University (ethics approval code is ORT-11-09-16A-14). All methods were carried out in accordance with relevant guidelines and regulations.

The articular cartilages were digested to isolate human articular chondrocytes (HACs) from the matrix by collagenase type II (Thermo Fisher Scientific, Dun Laoghaire, Co Dublin, Ireland) digestion for 24 h in Dulbecco’s Modified Eagle’s Medium (DMEM) at 37 °C. After washing with PBS, HACs were grown in DMEM with 10% (v/v) FCS as high-density primary monolayer cultures until confluent growth occurred.

### Measurement of matrix metalloproteinase expression

HACs at passage 4 (2.5 × 10^5^ cells/well) were cultured in 6 well plates. After 24 h starvation, cells were co-treated with either anthocyanins or metabolites (6.25–50 µg/ml) together with 10 µg/ml of AGEs for 24 h. Then both culture media and cell lysates were harvested for protein and mRNA expression analysis of *MMP-1*, *-3* and *-13*.

For examination of MMP gene expression, the total RNA from HACs was extracted using an Illustra RNAspin mini kit (GE Healthcare, Chicago, IL, USA) according to the manufacturer’s instructions. The cDNA was synthesized from 0.5 µg total RNA of each sample template via reverse transcription using a Tetro cDNA Synthesis Kit (Bioline, Alexandria, NSW, Australia). The gene expression of pro-inflammatory cytokines and MMPs degradation were examined by real-time quantitative polymerase chain reaction using the 7500 Fast real-time PCR system with SentiFASTTM SYBR Lo-ROX kit (Bioline, Alexandria, NSW, Australia). The reaction was performed with forty cycles: 95 °C 5 s, 60 °C 10 s and 72 °C 30 s of each cycle. GAPDH was used to normalize gene expression. The relative expression of each primer set was calculated by the 2(− ^ΔΔ^C(T)) method. The sequence of each of the primers used in RT-qPCR reaction was normalized in relative expression level by the *GAPDH* gene (Forward: 5′-AGGGCTGCTTTTAACTC TGGT-3′, Reverse: 5′-CCCCACTTGATTTTGGAGGGA-3′). Determination of AGEs induced HACs was done by analysis of *MMP-1* genes (Forward: 5′-CTGTTCAGGGACA GAATGTGCT-3′, Reverse: 5′-TCGATATGCTTCACAGTTCTAGGG), *MMP-3* genes (Forward: 5′-TTTTGGCCATCTCTTCCTTCA-3′, Reverse: 5′-TGTGGATGCCTCTT GGGTATC) and *MMP-13* genes (Forward: 5′-TCCTCTTCTTGAGCTGGACTCATT-3′, Reverse: 5′-CGCTCTGCAAACTGGAGGTC).

The protein levels of MMP-1, -3 and -13 in the culture medium were determined using a human MMP-1, -3 and -13 ELISA kit (R&D Systems, Minneapolis, MN, USA) following the manufacturer’s instructions. The protein concentrations were calculated according to the standard curve using the standard recombinant protein in the ELISA kits. The MMP concentrations were calculated according to the standard curve using the standard recombinant protein in the ELISA kits. The limits of quantification (LOQ) of MMP-1, -3 and -13 are 0.2 ng/ml, 0.2 ng/ml and 78 pg/ml, respectively.

### Signaling pathways western immunoblotting analysis

To investigate the molecular mechanism of purple corn anthocyanins and PCA on AGEs induced MAPK and NF-κB activation, the levels of phosphorylated signaling proteins were analyzed by Western blotting. The concentration of 2.5 × 10^5^ per well of HACs at passage 4 was cultured in 6-well plates. The 80% confluent of HACs was cultured without serum overnight and then pretreated with anthocyanins (C3G, Pg 3-glc and P3G) and PCA (6.25–50 µg/ml) for 2 h followed by stimulation with 10 μg/ml of AGEs for another 10 min. The cytoplasmic proteins of HACs were extracted using RIPA buffer and were separated using 12% (w/v) of SDS-PAGE. Proteins were transferred onto nitrocellulose membranes which were later blocked with 5% (w/v) non-fat dried-milk proteins in PBST. After blocking, various probes, including p65, p-p65, IκB-α, p38, p-p38, JNK, p-JNK, ERK, p-ERK or β-actin antibody (Cell Signaling Technology, Inc., Beverly, MA, USA) were added and incubated overnight. The membranes were then washed with PBST and HRP-conjugated secondary antibodies were added. Incubation was performed for 1 h and the bands were visualized using enhanced chemiluminescence (ECL) reagent (GE Healthcare, Chicago, IL, USA). The quantification of band intensity was performed by TotalLab TL120 v2006 (for Windows, TotalLab software, Newcastle upon Tyne, UK, www.nonlinear.com).

### Statistical analysis

Results are expressed as the average mean ± standard deviation from three independent experiments; each was run in triplicate (n = 3). One-way ANOVA was used to compare AGEs treatment alone and AGEs co-treatment with anthocyanin or its metabolite. Results were considered statistically significant when *p* < 0.05.

## Supplementary information

Supplementary Information
