# Supplementary material for: Chondroprotective effects of purple corn anthocyanins on advanced glycation end products induction through suppression of NF-κB and MAPK signaling
Source: Sci Rep. 2021 Jan 21;11:1895. doi: 10.1038/s41598-021-81384-4 (PMC7820347; doi:10.1038/s41598-021-81384-4)
Supplement: Supplementary file 1 — Supplementary Information [file 41598_2021_81384_MOESM1_ESM.doc]

**Chondroprotective effects of purple corn anthocyanins on advanced glycation end products induction through suppression of NF-κB and MAPK signaling**

Hathaichanok Chuntakaruk, Prachya Kongtawelert and Peraphan Pothacharoen*

Thailand Excellence Center for Tissue Engineering and Stem Cells, Department of

Biochemistry, Faculty of Medicine, Chiang Mai University, Chiang Mai 50200, Thailand

**
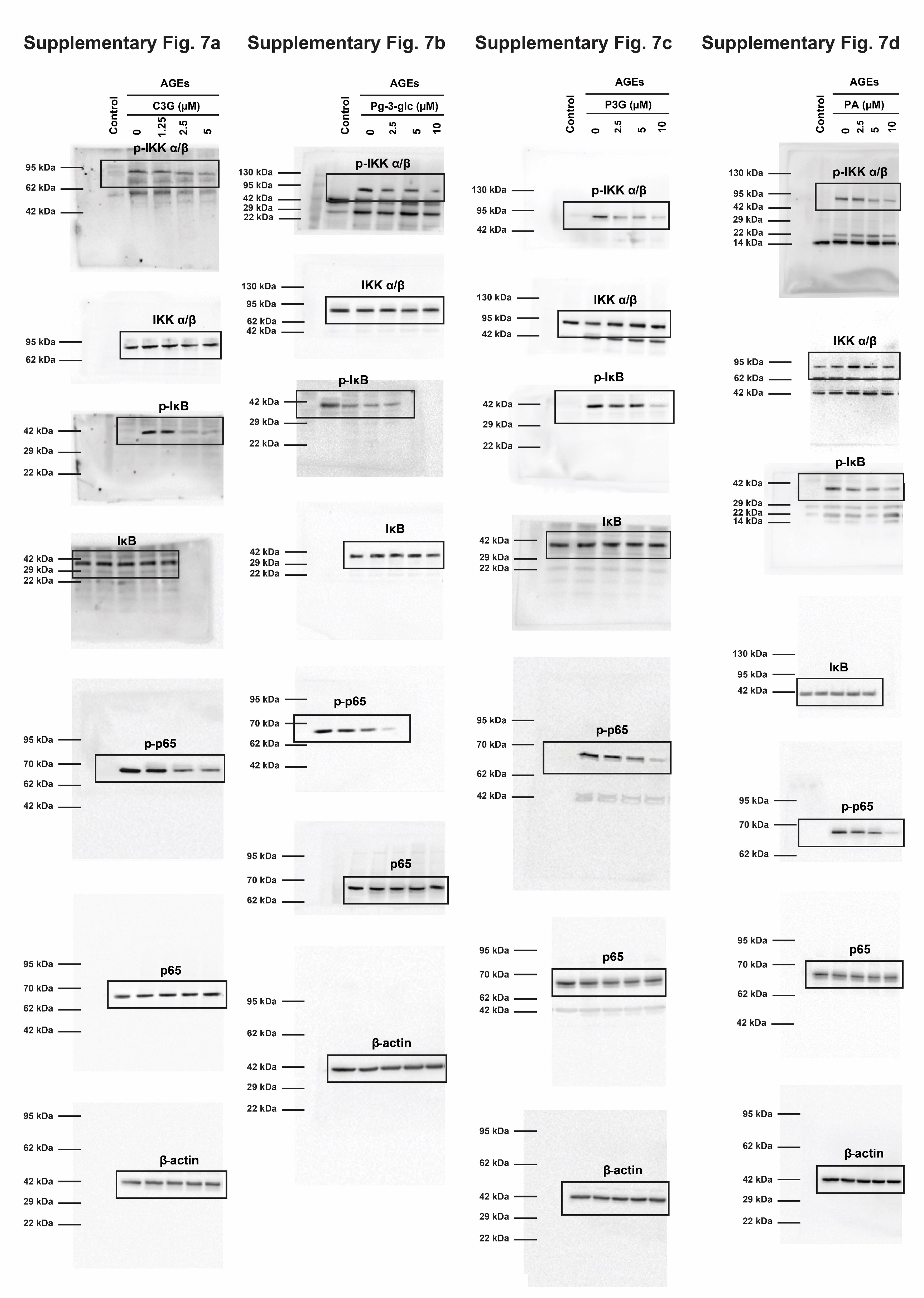
**

**Supplementary Figure 7**

Uncropped full-length pictures of Western blotting membranes presented in the Supplementary Fig. 7a-7d. Membranes were often cut to enable blotting for multiple antibodies.


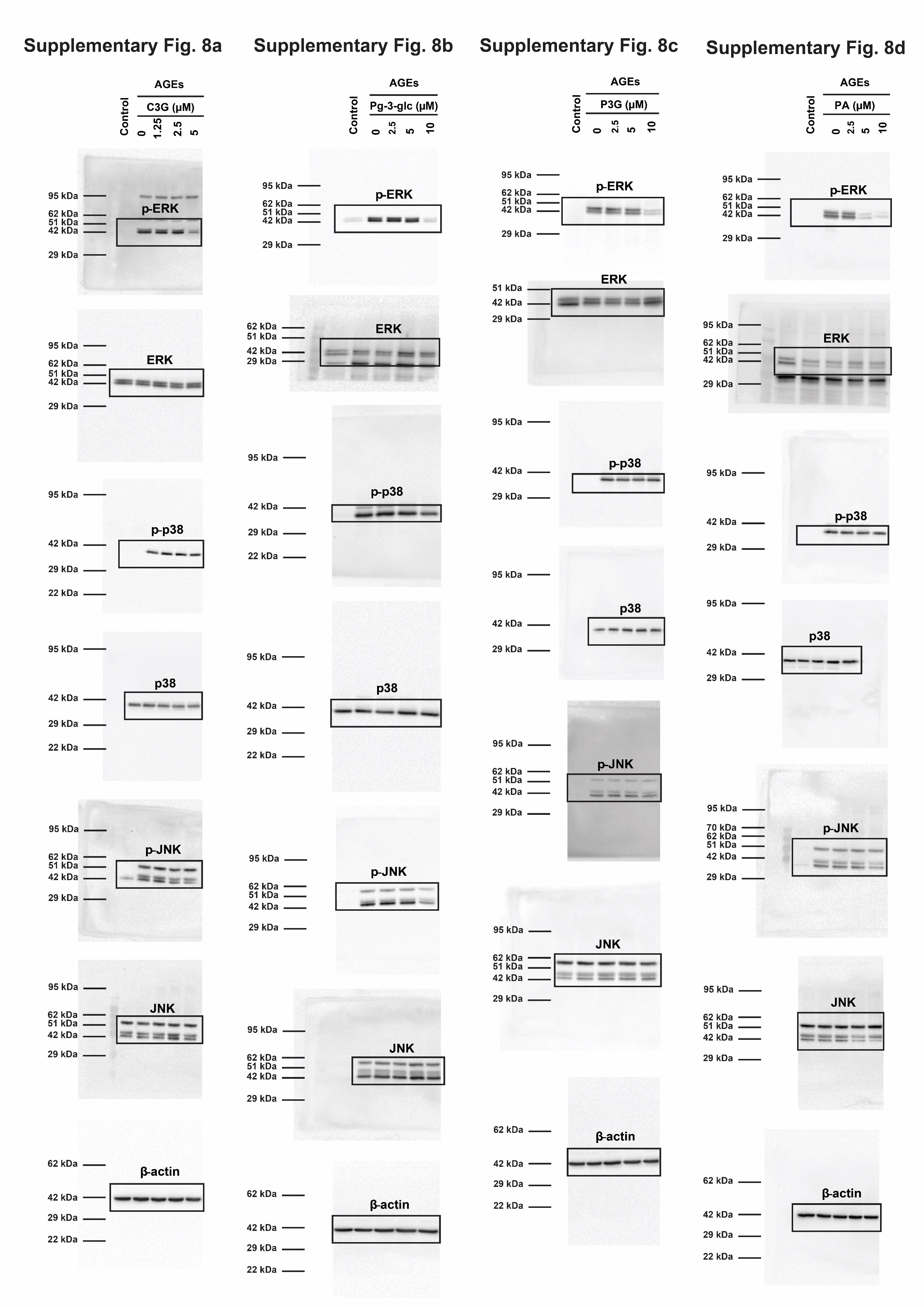


**Supplementary Figure 8**

Uncropped full-length pictures of Western blotting membranes presented in the Supplementary Fig. 8a-8d. Membranes were often cut to enable blotting for multiple antibodies.
